# Supplementary material for: Integrative Epigenetic and Molecular Analysis Reveals a Novel Promoter for a New Isoform of the Transcription Factor TEAD4
Source: Int J Mol Sci. 2024 Feb 13;25(4):2223. doi: 10.3390/ijms25042223 (PMC10888684; doi:10.3390/ijms25042223)
Supplement: Supplementary file 1 [file ijms-25-02223-s001.zip › ijms-2853249-supplementary.pdf]

Supplementary information

Integrative epigenetic and molecular analysis reveals a novel promoter for a new isoform of the transcription factor TEAD4

Shima Rashidiani [1], Gizaw Mamo [1], Benjámin Farkas [1], András Szabadi[1,2] ,Bálint Farkas [3,4], Veronika Uszkai [3], András Császár [3], Barbara Brandt [5], Kálmán Kovács [3,4], Marianna Pap [5,#] and Tibor A. Rauch [1,#,*]

1 Institute of Biochemistry and Medical Chemistry, Medical School, University of Pécs; 7624 Pécs, Hungary
2. Department of Dentistry, Oral and Maxillofacial Surgery, Medical School, University of Pécs, 7623 Pécs, Hungary
3. Department of Obstetrics and Gynecology, Medical School, University of Pécs, 7624 Pécs, Hungary;
4. National Laboratory of Human Reproduction, University of Pécs, 7624 Pécs, Hungary
5 Department of Medical Biology and Central Electron Microscope Laboratory, Medical School, University of Pécs; 7624 Pécs, Hungary
* Correspondence: tibor.rauch@aok.pte.hu
# Shared authors

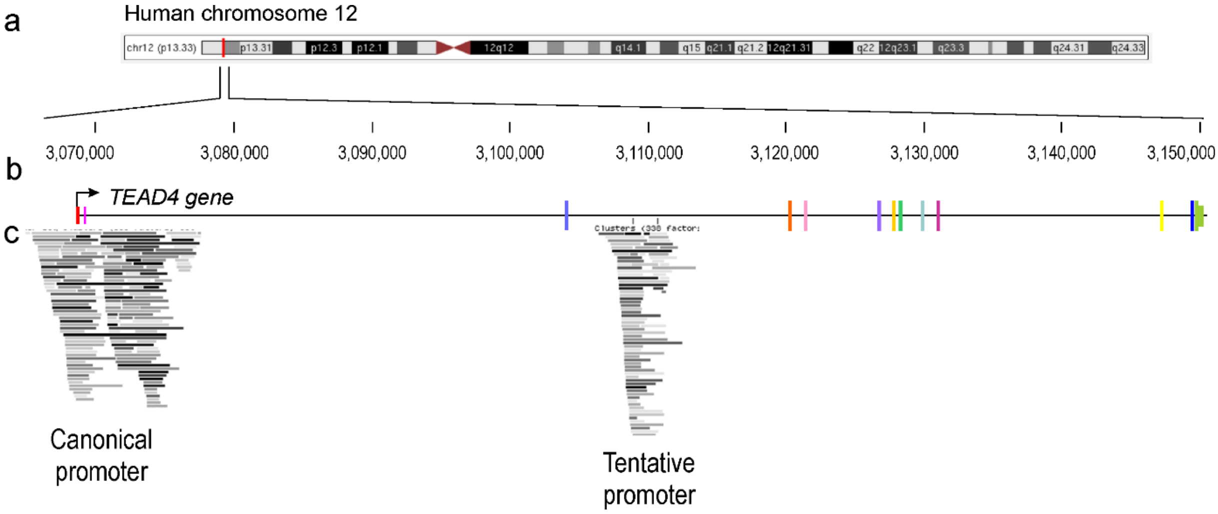

Figure S1. The transcription factor ChIP-Seq data in the TEAD4 locus. (a) The TEAD4 gene is located on the left arm of human chromosome 12, as indicated by the vertical red line. The exon/intron structure of the TEAD4 gene is shown in (b) with colored vertical lines representing exons and thin horizontal lines representing intronic regions. (c) The location of ChIP-Seq data for specific transcription factors is represented by gray horizontal lines.

Supplementary information

>TEAD4-ΔN

CCAGCCCTCCCCTTGAGCACCTCTTACCCTCCTGGCCGGCTCCCGAGCCCGGGGTGCTTGCCTTCTCCTGCT
CACGGCCGCTTTCATTTCTGCCCTGTCACTGTGTGACCCTCCCCCTGGCACAGCCAACGCTGGCCACCCTGAC
CTCCTTTGGCCAGGCTCACAGTCGGCCTAGCCTAGCGTGCAGGTCGGAACGAGCTGATTGCCCGCTACATCAA
GCTCCGGACAGGGAAGACCCGCACCAGGAAGCAGGTCTCCAGCCACATCCAGGTGCTGGCTCGTCGCAAAGCT
CGCGAGATCCAGGCCAAGCTAAAGGACCAGGCAGCTAAGGACAAGGCCCTGCAGAGC**ATGGCTGCCATGTCGT**
CTGCACAGATCATCTCCGCCACGGCCTTCCACAGTAGCATGGCCCTCGCCCGGGGCCCCGGCCGCCCAGCAGT
CTCAGGGTTTTGGCAAGGAGCTTTGCCAGGCCAAGCCGGAACGTCCCATGATGTGAAGCCTTTCTCTCAGCAA
ACCTATGCTGTCCAGCCTCCGCTGCCTCTGCCAGGGTTTGAGTCTCCTGCAGGGCCCGCCCCATCGCCCTCTG
CGCCCCCGGCACCCCCATGGCAGGGCCGCAGCGTGGCCAGCTCCAAGCTCTGGATGTTGGAGTTCTCTGCCTT
CCTGGAGCAGCAGCAGGACCCGGACACGTACAACAAGCACCTGTTCGTGCACATTGGCCAGTCCAGCCCAAGC
TACAGCGACCCCTACCTCGAAGCCGTGGACATCCGCCAAATCTATGACAAATTCCCGGAGAAAAAGGGTGGAC
TCAAGGATCTCTTCGAACGGGGACCCTCCAATGCCTTTTTTCTTGTGAAGTTCTGGGCAGACCTCAACACCAA
CATCGAGGATGAAGGCAGCTCCTTCTATGGGGTCTCCAGCCAGTATGAGAGCCCCGAGAACATGATCATCACC
TGCTCCACGAAGGTCTGCTCTTTCGGCAAGCAGGTGGTGGAGAAAGTTGAGACAGAGTATGCTCGCTATGAGA
ATGGACACTACTCTTACCGCATCCACCGGTCCCCGCTCTGTGAGTACATGATCAACTTCATCCACAAGCTCAA
GCACCTCCCTGAGAAGTACATGATGAACAGCGTGCTGGAGAACTTCACCATCCTGCAGGTGGTCACCAACAGA
GACACACAGGAGACCTTGCTGTGCATTGCCTATGTCTTTGAGGTGTCAGCCAGTGAGCACGGGGCTCAGCACC
ACATCTACAGGCTGGTGAAAGAATGAGAGACTCGGGGAGCAGGGAGGGGGGAAG

Figure S2. The primary nucleotide sequence of the mRNA encoding the truncated TEAD4 isoform. The sequence contains alternating exons, indicated by yellow and gray shading. The novel (non-coding) exon sequence is underlined in red, while the open reading frame (ORF) is marked in bold.

Supplementary information

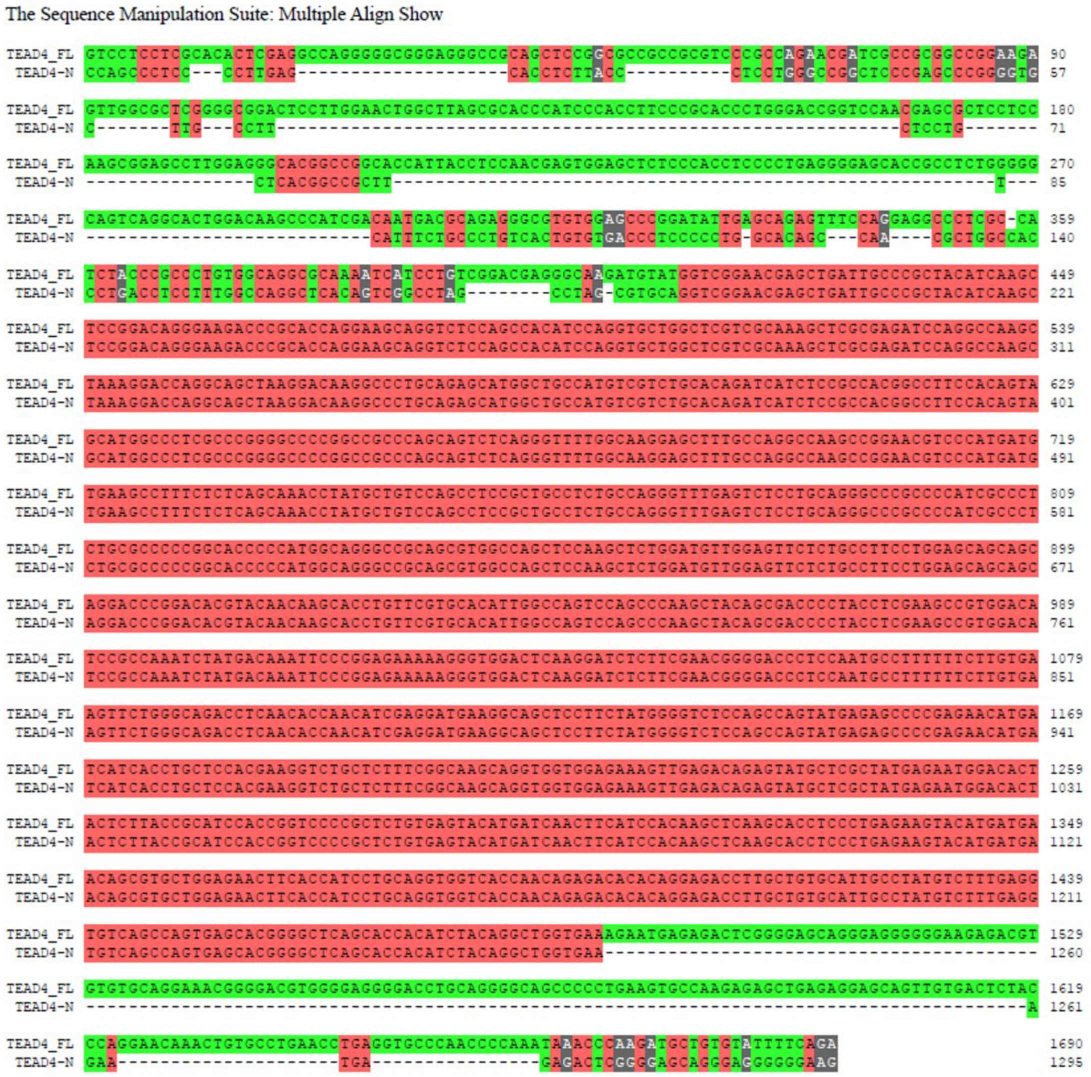

Figure S3. Pairwise alignment of mRNAs encoding TEAD4 isoforms. The nucleotide sequences of the full-length coding transcript of TEAD4 and the newly identified TEAD4-ΔN isoform encoding transcript are aligned using . The nucleotides highlighted in red are identical residues.

Supplementary information

Analysis	Name	Forward/Reverse	Notes
**5'RACE**	Anchor_F1	GACCACGCGTATCGATGTCGACTTTTT TTTTTTTTTTTV	
	Anchor_F2	GTATCGATGTCGACTTTTTTTTTT	
	GenSpec_RT	ACTCAAACCCTGGCAG	Reverse transcription
	GenSpec_R1	CATGGGACGTTCCGGC	
	GenSpec_R2	AAGCTCCTTGCCAAAAC	
**Gene expr.**	TEAD4-C_F	TGAGCAGAGTTTCCAGGAGG	End-point PCR
	TEAD4-C_R	CCTTGCCAAAACCCTGAGAC	
	TEAD4-del_F	TCCCCTTGAGCACCTCTTAC	End-point PCR
	TEAD4-del_R	TCTCAGGGTTTTGGCAAGGA	
**Promoter cloning**	TEAD-PromC_oF	TCATGCACTTCCCTATCCCA	Nested PCR
	TEAD-PromC_oR	TGAGGGCTGGAATGAGATGC	
	TEAD-PromC_InF	aactcgagCCACCCAACACAAGGCTAAG	
	TEAD-PromC_InR	aaaagcttGGCTGGAATGAGATGCGC	
	TEAD-PromAlt_oF	TCTCTTTGGCAGGAGGGAAG	Nested PCR
	TEAD-PromAlt_oR	CAGGAGGGTAAGAGGTGCTC	
	TEAD-PromAlt_inF	aactcgagCAGTCTGCTTCCCTGAGTCA	
	TEAD-PromAlt_inR	aaaagcttGTTAATCTGCCGTGCTGCTT	
	Prom-Seq_F	gtacgggaggtacttggagcg	Sanger Seq
	Prom-Seq_R	ccacctcgatatgtgcatctgtaaaa	
**Fusing cDNAs to FP**	TEAD4-EGFP_F	aaaaagcttGCC**ATG**GAGGGCACGGC	TTG mutated to ATG to increase translation
	TEAD4-EGFP_R	aa*ggatcc*TTCTTTCACCAGCCTGTA	
	TEAD4-RFP_F	**aaaagcttATG**GCTGCCATGTCGTCTG	
	TEAD4-RFP_R	aa*ggatcc*TTCTTTCACCAGCCTGTA	
	EGFP-Seq_F	CCTCGCCCTCGCCGGA	Sanger Seq
	RFP-Seq_F	GCCCTCGTAGGGCTTGC	
**DNA methylation anal.**	BS_PromC_F	ATGGTATGTTATATGGATTTTAAAT	
	BS_PromC_R	CAAAAAAAACTACTCCCTTCCTAC	
	BS_PromAlt_F	TAGGTTTTGTTGGGATTTAGATATT	
	BS_PromAlt_R	CATCCCACACACTAAAAATAAACTC	
	T7	GCCAAGCTCTAATACGACTCACTATA GG	Sanger Seq
	Sp6	GCCATTTAGGTGACACTATAGAATAC	

Table S1. Oligonucleotides used in end-point PCR, cloning and DNA methylation (i.e., Bisulfite Sequencing) analysis

Supplementary information

Analysis	Name	Forward/Reverse	Notes
EMSA	TEAD4-ΔN-Bio_F	CCCTCTGGGAGGCGGAATGGGAGGCCGAGCTG	5'-biotyn
	TEAD4-ΔN_F	CCCTCTGGGAGGCGGAATGGGAGGCCGAGCTG	
	TEAD4-ΔN_R	CAGCTCGGCCTCCCATTCCGCCTCCCAGAGGG	
	TEAD4-ΔN-Mut_Bio_F	CCCTCTGGGAGGCGGACGGGGAGGCCGAGCTG	5'-biotyn
	TEAD4-ΔN-Mut_F	CCCTCTGGGAGGCGGACGGGGAGGCCGAGCTG	
	TEAD4-ΔN-Mut_R	CAGCTCGGCCTCCCCGTCCGCCTCCCAGAGGG	
	TEAD4-cons-F-Bio	CGGCGATGTGACCTGGAATGTGGCGTCCGTAT	5'-biotyn
	TEAD4-cons-F	CGGCGATGTGACCTGGAATGTGGCGTCCGTAT	
	TEAD4-cons-R	ATACGGACGCCACATTCCAGGTCACATCGCCG	

Table S2. Oligonucleotides for DNA-protein interaction studies (EMSAs). The corresponding complementary reverse oligos were annealed to the 5'-biotinylated oligos. In competition assays, un-biotinylated forward were annealed and used in essays with varying molar concentrations.
